# Detection of Bone Marrow Edema in Patients with Osteoid Osteoma Using Three-Material Decomposition with Dual-Layer Spectral CT

**DOI:** 10.3390/diagnostics11060953

**Published:** 2021-05-26

**Authors:** Florian T. Gassert, Johannes Hammel, Felix C. Hofmann, Jan Neumann, Claudio E. von Schacky, Felix G. Gassert, Daniela Pfeiffer, Franz Pfeiffer, Marcus R. Makowski, Klaus Woertler, Alexandra S. Gersing, Benedikt J. Schwaiger

**Affiliations:** 1 Department of Radiology, Klinikum Rechts der Isar, School of Medicine, Technical University of Munich, 81675 Munich, Germany; johannes.hammel@tum.de (J.H.); felix.hofmann@tum.de (F.C.H.); jan.neumann@tum.de (J.N.); claudio.vschacky@gmail.com (C.E.v.S.); felix.gassert@tum.de (F.G.G.); daniela.pfeiffer@tum.de (D.P.); franz.pfeiffer@tum.de (F.P.); marcus.makowski@tum.de (M.R.M.); klaus.woertler@tum.de (K.W.); alexandra.gersing@tum.de (A.S.G.); 2 Chair of Biomedical Physics, Technical University of Munich, 85748 Garching, Germany; 3 Musculoskeletal Tumor Center, Klinikum Rechts der Isar, School of Medicine, Technical University of Munich, 81675 Munich, Germany; 4 Department of Neuroradiology, University Hospital Munich, Ludwig Maximilian University of Munich, 80336 Munich, Germany; 5 Department of Neuroradiology, Klinikum Rechts der Isar, School of Medicine, Technical University of Munich, 81675 Munich, Germany; benedikt.schwaiger@tum.de

**Keywords:** dual-layer spectral computed tomography, dual-energy CT, osteoid osteoma, bone marrow edema

## Abstract

The aim of this study is to assess whether perifocal bone marrow edema (BME) in patients with osteoid osteoma (OO) can be accurately detected on dual-layer spectral CT (DLCT) with three-material decomposition. To that end, 18 patients with OO (25.33 ± 12.44 years; 7 females) were pairwise-matched with 18 patients (26.72 ± 9.65 years; 9 females) admitted for suspected pathologies other than OO in the same anatomic location but negative imaging findings. All patients were examined with DLCT and MRI. DLCT data was decomposed into hydroxyapatite and water- and fat-equivalent volume fraction maps. Two radiologists assessed DLCT-based volume fraction maps for the presence of perifocal BME, using a Likert scale (1 = no edema; 2 = likely no edema; 3 = likely edema; 4 = edema). Accuracy, sensitivity, and specificity for the detection of BME on DLCT were analyzed using MR findings as standard of reference. For the detection of BME in patients with OO, DLCT showed a sensitivity of 0.92, a specificity of 0.94, and an accuracy of 0.92 for both radiologists. Interreader agreement for the assessment of BME with DLCT was substantial (weighted κ = 0.78; 95% CI, 0.59, 0.94). DLCT with material-specific volume fraction maps allowed accurate detection of BME in patients with OO. This may spare patients additional examinations and facilitate the diagnosis of OO.

## 1. Introduction

Osteoid osteoma (OO) accounts for about 5% of all bone tumors, and 10% of benign bone tumors [1]. It more commonly affects males, with an approximate male/female ratio of 2:1, and is usually found in children, adolescents, and young adults, between the ages of 10 and 35 years. The most common symptom is nocturnal pain that responds well to nonsteroidal anti-inflammatory drugs [2]. The final diagnosis is made by imaging in consensus with the clinical findings. The imaging method of choice in patients with suspected OO is CT, as not only can the exact localization of the tumor be identified, but also the main imaging features of the OO can be depicted: a small round to oval hypodense area (“nidus”) ≤2 cm, surrounded by bone sclerosis [3]. While CT allows superior depiction of the bone structure, MR imaging is more sensitive for bone marrow edema (BME) as an essential imaging finding in the diagnosis of OO, which correlates with the presence of clinical symptoms. In cases where a CT was performed for an unclear condition and a nidus-like lesion was found as an incidental finding, MR imaging was often used to rule out BME, and therefore, the diagnosis of an active OO. Furthermore, the diagnostic workup of patients with clinically suspected recurrence of OO after therapy includes both CT for the detection of a potential nidus and MR imaging for the detection of the BME. However, MR imaging generates additional costs and examination time, and patients may have contraindications for the examination. Therefore, alternatives to MR imaging for visualizing the BME surrounding the OO would be helpful, and optimally, both the nidus and the presence of BME could be depicted in one single examination. 

Several studies have shown that dual-energy CT has a higher sensitivity for BME compared to conventional CT [4,5,6,7]. Furthermore, a recent study showed that three-material decomposition using data from dual-layer spectral CT enables the detection of BME in patients with acute vertebral fractures [8]. Thus, dual-layer spectral CT may be promising for the visualization of BME in patients with OO.

The aim of this study was to evaluate whether three-material decomposition with dual-layer spectral CT allows for the detection of BME in patients with OO and whether spectral CT could replace the combination of conventional CT and MR examinations in those patients.

## 2. Methods

### 2.1. Patient Selection

**Figure 1:**
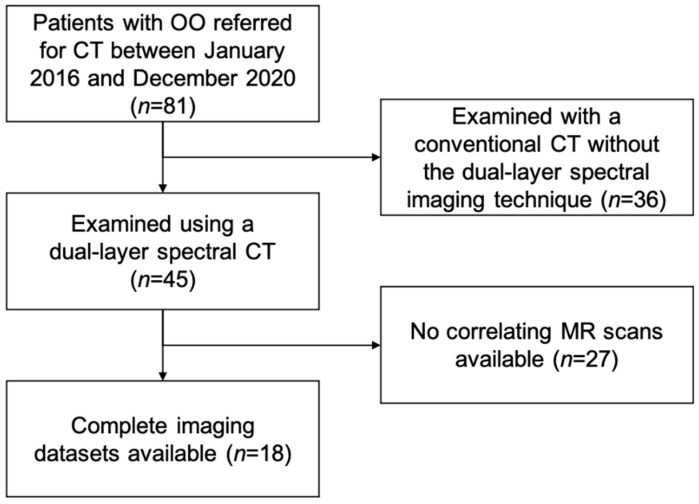
Flowchart illustrating patient selection.

Institutional review board approval was obtained prior to this study Ethics Commission of the Medical Faculty, Technical University of Munich, Germany). Written informed consent was waived for this retrospective analysis of routinely acquired imaging data. In our institutional Picture Archiving and Communication System (PACS), patients with OO referred for CT between January 2016 and December 2020 were retrospectively identified (*n* = 81). The diagnosis was verified by an expert panel of radiologists (F.T.G., J.N., A.S.G., with 4, 8, and 12 years of experience in MSK imaging, respectively) and orthopedic surgeons based on clinical and imaging findings. Of the 81 potential participants, 45 were examined on one dual-layer spectral CT scanner, and of those, 18 had a recent MR examination of the same region with fluid-sensitive sequences (Figure 1). All cases examined using the dual-layer spectral CT were part of the diagnostic work up of the primary diagnosis of an osteoid osteoma; none of the cases showed a recurrent osteoid osteoma. These patients were matched to 18 patients that had also undergone both dual-layer spectral CT and MR imaging as part of their clinical workup at corresponding anatomic sites but showed unremarkable findings (control group). Indications for CT imaging in the control group were soft tissue masses (*n* = 7), unclear pain without any previous pathological imaging findings (*n* = 3), cartilage defects (*n* = 2), recurrent shoulder dislocation (*n* = 2), impingement (*n* = 1), a suspected fracture (*n* = 1), a wound healing disorder (*n* = 1), and a suspected hematoma (*n* = 1). The absence of pathologic imaging findings in those patients was again verified by an expert panel of radiologists.

### 2.2. CT-Imaging Protocol. and Post-Processing

CT images were acquired using a dual-layer spectral CT scanner (IQon Spectral CT, Philips Healthcare, Hamburg, Germany).

Patient examinations were performed with a tube voltage of 120 kVp and exposures varying between 34.0 and 227.0 mAs, with an average exposure of 92.1  ±  40.8 mAs. The resulting CT dose indices (CTDIvol) recorded in the dose reports generated by the scanner ranged from 3.2 to 19.4 mGy, with an average of 8.4  ±  3.6 mGy. Spectral base image (SBI) datasets were reconstructed using a bone reconstruction kernel with an axial slice thickness of 0.9 mm. To homogenize images and reduce noise, sagittal reformations were averaged up to a thickness of 5 mm.

SBI datasets contain information on energy-dependent absorption, extracted with dual-layer detector technology. This information can be used to create virtual monochromatic images at 50 and 200 keV, showing absorption information on a HU scale as if acquired from a monochromatic X-ray source [9]. These images were generated with IntelliSpace Portal (version 11.1.1, Philips Healthcare, Hamburg, Germany).

For further image calculations, an advanced post-processing platform (IntelliSpace Discovery 3.0.6, Philips Healthcare) was used. An in-house-developed plug-in was applied to generate red marrow/water, yellow marrow/fat, and hydroxyapatite volume fraction maps by applying a three-material decomposition on the spectral information from the monochromatic images (plug-in available from the authors on request), i.e., generating material-specific “density maps”. Interest lies on the red marrow map, as it contains more than 90% of water and therefore produces results comparable to fluid-sensitive MR imaging sequences, such as short-tau inversion recovery (STIR) [8].

Dual-layer CT, like other dual-energy methods, utilizes the assumption of being able to split the attenuation coefficient into two physically relevant absorption processes: the photo effect and Compton scattering. A decomposition in photo and Compton coefficient is resolvable for a spectrally separated measurement. Assuming this, the generation of only two linear independent virtual monochromatic images can be performed. To decompose the spectral dataset into three volume fractions, it is necessary to introduce a third equation. It is assumed that the volume of a material mixture at a given temperature and pressure equals the sum of the volume of its constituent parts at the same temperature and pressure [10]. Mathematically, the sum of the volume fractions needs to be equal to one. Spectral image analysis algorithms, like the generation of virtual non-enhanced images and liver-fat quantification, apply this or similar assumptions.

Hounsfield units, the entity in monochromatic images, are converted to attenuation coefficients using the formula:

$$\mu \left(E\right)=\left(HU\left(E\right)/1000\;\left[HU\right]\right)\times \mu_{Water}\left(E\right)+\mu_{Water}\left(E\right)$$

with $\mu_{Water}\left(E\right)$ being the attenuation coefficient of water at energy E obtained from the United States National Institute of Technology and Standards (NIST) X-ray Mass Attenuation Coefficients Database [11]. HU(E) equals the virtual monochromatic image being calculated from energy E using the IntelliSpace Portal. The three-material decomposition can be expressed as linear equation system, where we solve for volume fractions *f*_1_, *f*_2_, and *f*_3_ of red marrow, yellow marrow, and hydroxyapatite in image space.

$$\mu \left(E_{1}\right)=f_{1}\mu_{1}\left(E_{1}\right)+f_{2}\mu_{2}\left(E_{1}\right)+f_{3}\mu_{3}\left(E_{1}\right)\;\mu \left(E_{2}\right)=f_{1}\mu_{1}\left(E_{2}\right)+f_{2}\mu_{2}\left(E_{2}\right)+f_{3}\mu_{3}\left(E_{2}\right)\;f_{1}+f_{2}+f_{3}=1$$

μ(E) is the measured attenuation coefficient at energy *E*_1_ (set to 50 keV) and *E*_2_ (set to 200 keV). $\mu_{1,2,3}\left(E\right)$ are the attenuation coefficients of red marrow, yellow marrow, and hydroxyapatite in pure form. The International Commission on Radiation Units and Measurements Report 46 [12] gives theoretically calculated values for the chemical composition of those materials. In the final step, the linear equation system needs to be solved for every pixel in our 3D volume. The computational process for solving the equation system in a C# loop for approximately 50 million times takes about 30 s on the IntelliSpace Discovery Server.

Material density maps can be generated by multiplying the volume fraction maps with the basis material density in pure form:

$$\rho_{1,2,3}=f_{1,2,3}\times \rho_{1,2,3\;pure}$$

This was done to obtain hydroxyapatite specific density maps.

Material maps for red marrow/water, yellow marrow/fat, and hydroxyapatite volume fractions were generated in axial, coronal, or sagittal orientation and a section thickness of 3 mm. In addition to grayscale reformations, color-coded variants of volume fraction maps were calculated with a standard rainbow filter within the IntelliSpace Discovery software suite transferring whiter grayscale values to warmer colors (Figure 1). As part of the standard-of-care protocol, conventional polychromatic CT images identical to routine MDCT images were generated and reformatted in a section thickness of 3 mm, based on a standard bone filter (YB). CT imaging data post-processing took around 3.1 ± 1.2 min for each examination.

### 2.3. MRI Acquisition

MRI were acquired with a minimum of two different planes. All MR acquisitions were conducted using a STIR sequence. Imaging was performed at either 1.5 T or 3.0 T MRI systems. Imaging parameters varied depending on the scanned location. Typical imaging parameters for 3.0 T were 4500 ms repetition time, 47 ms echo time, 156° flip angle, 180 mm field of view, 4 mm slice thickness, 25% gap, 252 Hz/pixel bandwidth, and 210 ms inversion time. Typical imaging parameters for 1.5 T were 3470 ms repetition time, 31 ms echo time, 150° flip angle, 200 mm field of view, 4 mm slice thickness, 50% gap, 161 Hz/pixel bandwidth, and 150 ms inversion time.

### 2.4. Image Analysis

MR images were assessed by an expert panel of radiologists (F.T.G., J.N., A.S.G., with 4, 8, and 12 years of experience in MSK imaging, respectively) and used as the standard of reference for the presence of BME. For this, BME was defined as an ill-defined area of hyperintense signal intensity on fluid-sensitive MR images, which on corresponding T_1_-weighted images showed decreased signal intensity compared to marrow fat, but higher signal intensity than skeletal muscle. BME was seen in all 18 patients with OO and in none of the patients from the control group. The maximum extent of BME on MR images of patients with OO was measured by the expert panel in a consensus reading. In the same session the nidus size was measured on CT examinations and the location of the OO was classified using the following parameters:

- tubular bones: epiphyseal/metaphyseal/diaphyseal; spine: vertebral body, posterior elements
- intramedullar/cortical/subperiostal
- extra-/intra-articular

Material-specific volume fraction maps derived from dual-layer spectral CT images were independently assessed by two radiologists that were not part of the expert panel (F.G.G., B.J.S., with 4 and 12 years of experience in MSK imaging, respectively), blinded to clinical information and MRI findings, with a gap of at least four weeks to MRI readings and in a randomized order. The radiologists used only red marrow/water and yellow marrow/fat volume fraction maps and were allowed to scroll through the reformations and to adjust the window and level settings for personal convenience. A four-point Likert scale was used to describe the individual assessment of presence of BME (1 = no edema; 2 = likely no edema; 3 = likely edema; 4 = edema). Categories 1 and 2 were summarized as no presence of edema, and 3 and 4 as presence of BME. Additionally, both radiologists measured the maximum extent of edema in red marrow/water maps.

On CT imaging, diagnostic image quality was rated on a scale from 1 to 5 (1 = not diagnostic, 2 = poor, 3 = good, 4 = very good, 5 = excellent).

### 2.5. Statistical Analysis

Statistical analyses were performed by F.T.G. with SPSS Version 24 (IBM, Armonk, NY, USA). A *p*-value of less than 0.05 was considered to indicate a statistically significant difference.

In addition to descriptive statistics, sensitivity, specificity, and accuracy for the detection of the presence of BME in DLCT images were assessed with MR imaging as standard of reference. The interreader reproducibility for DLCT imaging Likert scales was assessed with Cohen’s weighted κ. The interreader reproducibility for measurements of the BME extent was assessed with the intraclass correlation coefficient (ICC). The correlation of the measurements of maximum BME extent in DLCT images with MR based measurements as standard of reference was calculated using Pearson’s correlation coefficient.

## 3. Results

### 3.1. Patients and OO Characteristics

A total of 36 patients were included in this study (16 female, 26.03 ± 10.99 years). Between patients with and without OO, no significant differences were found regarding age (patients with OO, 25.33 ± 12.44 years versus patients without OO, 26.72 ± 9.65; *p* = 0.71) and sex (patients with OO, 7 females (38.89%) vs. patients without OO, 9 females (50.00%); *p* > 0.52). OO were located in the proximal femur (*n* = 7), hand (*n* = 4), spine (*n* = 2), clavicle (*n* = 2), and foot, radius, and tibia (*n* = 1, respectively). Table 1 shows the distribution of the localization of OO. The average maximum extent of BME as measured by the expert panel was 37.83 ± 27.43 mm. The average nidus size was 4.50 ± 1.25 mm.

### 3.2. DLCT

**Figure 2:**
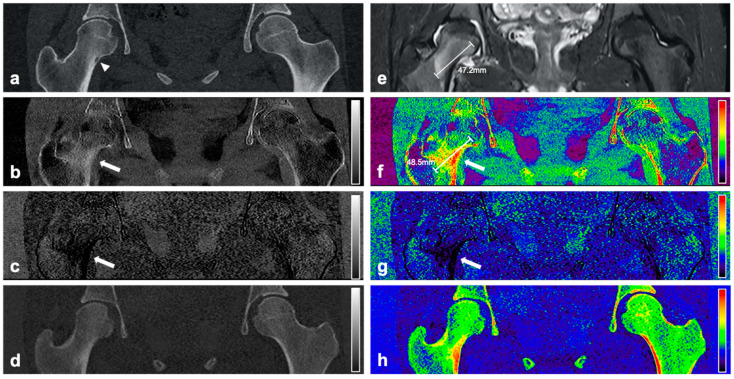
CT and MR images of an 18-year-old female patient with intracortical OO in the right femoral neck. Coronal reformation of the conventional CT examination (**a**) reveals the nidus in the right femoral neck with surrounding sclerosis (arrowhead). Material-specific volume fraction maps for red marrow/water (**b**), yellow marrow/fat (**c**), and hydroxyapatite (**d**), and respective color-coded overlays (**f**–**h**) reveal an increase in water-specific volume fraction in the red-marrow map (**b**,**f**; arrow) and a decrease in fat-specific volume fraction in the yellow-marrow map (**c**,**g**; arrow) in the right femoral neck, compared to the respective area on the other side. The corresponding coronal STIR MR image confirms the presence of a half-moon shaped edema-equivalent signal alteration in the right femoral neck around (“half-moon sign” [13]) (**e**). Calipers indicate measurements of the extent of BME.

**Figure 3:**
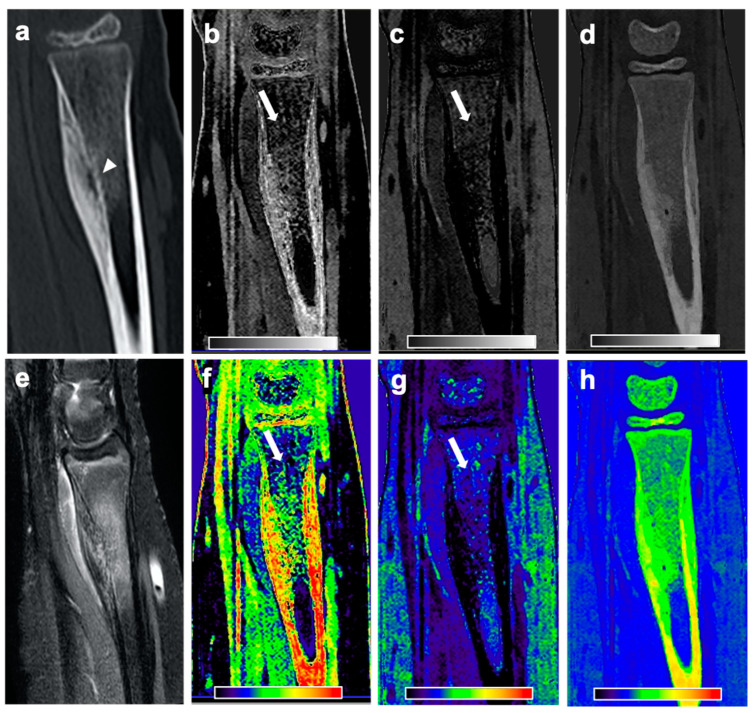
CT and MR images of a 12-year-old female patient with subperiosteal OO in the right distal radius. Sagittal CT reformation image (**a**) reveals the nidus in the right distal radius with surrounding sclerosis and a solid periosteal reaction (arrowhead). Material-specific volume fraction maps for red marrow/water (**b**), yellow marrow/fat (**c**), and hydroxyapatite (**d**), and respective color-coded overlays (**f**–**h**) reveal an increase in water-specific volume fraction in the red-marrow map (**b**,**f**; arrow) and a decrease in fat-specific volume fraction in the yellow-marrow map (**c**,**g**; arrow) in the right femoral neck, compared to the respective area on the other side. The corresponding sagittal STIR MR image confirms the presence of an edema-equivalent signal alteration in the bone marrow around the nidus (**e**).

**Figure 4:**
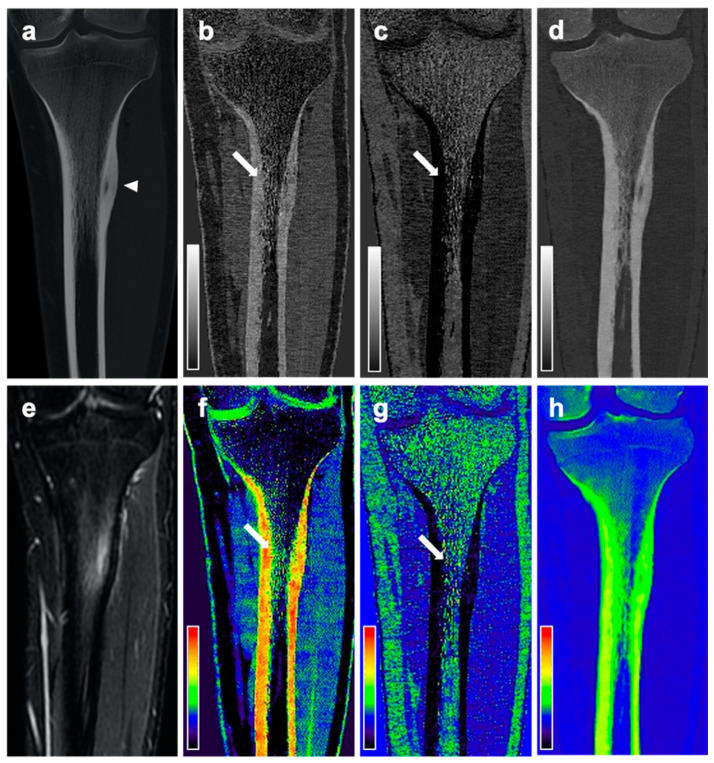
CT and MR images of a 19-year-old male patient with intracortical OO in the left tibia diaphysis. Coronal reformation of the conventional CT examination (**a**) reveals the nidus in the left tibia diaphysis (arrowhead). Material-specific volume fraction maps for red marrow/water (**b**), yellow marrow/fat (**c**), and hydroxyapatite (**d**), and respective color-coded overlays (**f**–**h**) reveal an increase in water-specific volume fraction in the red-marrow map (**b**,**f**; arrow) and a decrease in fat-specific volume fraction in the yellow-marrow map (**c**,**g**; arrow). The corresponding coronal STIR MR image confirms the presence of an edema-equivalent signal alteration in the left tibia diaphysis (**e**).

On DLCT-based volume fraction maps, radiologist 1 considered BME to be present in 16 of 18 patients (89%) with OO, and radiologist 2 found BME to be present in 17 of 18 patients (94%) with OO. In the controls, BME was found in 0% (radiologist 1) and 11% (radiologist 2) of the patients. Over all subjects, the mean Likert scale scores for the assessment of the presence of a BME on DLCT-based volume fraction maps were 2.39 ± 1.44 for radiologist 1, and 2.44 ± 1.40 for radiologist 2. In patients with OO, the mean Likert scale score was 3.67 ± 0.84 and 3.61 ± 0.70, respectively, while in controls, the mean Likert scale scores were 1.11 ± 0.32 and 1.28 ± 0.83, respectively. Figure 2, Figure 3 and Figure 4 show exemplary volume fraction maps and MR imaging of patients with OO. The average maximum extent of BME as measured by the readers on DLCT-based volume fraction maps was 32.89 ± 23.60 (30.89 ± 20.42 for radiologist 1, and 34.61 ± 21.83 for radiologist 2). Measurements of the maximum extent of BME on DLCT highly correlated with measurements on MRI (r = 0.96; *p* < 0.001).

### 3.3. Diagnostic Performance and Agreement

DLCT images showed a high sensitivity of 0.92 (95% confidence interval (CI): 0.72; 1.00), a specificity of 0.94 (0.74; 1.00), and an accuracy of 0.93 (0.73; 1.00) for the detection of BME for both radiologists separately (Table 2).

The average overall imaging quality score for DLCT based volume fraction maps was 4.10 ± 0.86. Interreader agreement for the assessment of BME on DLCT images using a four-point Likert scale was substantial (weighted κ = 0.78; 95% CI, 0.59, 0.94). The ICC for the assessment of the extent of BME on DLCT images was 0.81 (95% CI, 0.58, 0.87).

## 4. Discussion

In our study, three-material decomposition and material-specific volume fraction maps obtained from dual-layer spectral CT enabled the accurate detection of perifocal BME in patients with OO. Diagnostic performance for the detection of BME on DLCT was almost perfect compared to fluid-sensitive MR imaging sequences as standard of reference. Additionally, the correlation of measurements of the maximum extent of BME in DLCT and in MR imaging was high.

The diagnosis of OO according to the WHO criteria was based on clinical and imaging features [1]. CT is the modality of choice for diagnosing and characterizing OO, with the key findings being a well-defined round or oval lytic bone lesion (nidus) surrounded by reactive sclerosis [14]. MR imaging may also be able to depict the nidus, but its sensitivity greatly depends on the location of the lesion. Intramedullar OO are easier to detect on MR imaging compared to those within or close to cortical bone, and CT has shown to be more specific for identifying a nidus independent of its location [15,16]. Therefore, if clinical features are suggestive, CT should be the first imaging modality to be performed [17].

Nevertheless, in clinical practice, MR imaging is often the first imaging modality performed, due to the predominantly young age of the affected patients. However, in patients suspicious of recurrence following CT-guided ablation or surgical resection of OO, CT is the imaging modality of choice. In such cases, additional MR imaging is often performed for perifocal BME. BME is one of the key features of an active OO and its absence usually precludes local recurrence. DECT has gained more attention with regard to the detection of BME, as it allows for both a detailed depiction of osseous pathologies and the assessment of BME. In patients with OO, this allows detection of the nidus, evaluation of the presence and extent of bone sclerosis, and detection of BME in one examination. This may be very useful for the primary diagnosis of OO, but also for the detection of post-therapeutic recurrence. However, there are other imaging options available for unclear cases, one of which is dynamic contrast enhanced imaging [18,19]. A study by Liu et al. showed that osteoid osteomas can be imaged with greater conspicuity by using dynamic gadolinium-enhanced instead of nonenhanced MR imaging and also with equal or even more prominent conspicuity than on CT [20].

There are different ways to generate spectral information from CT imaging data, and therefore, there are different ways to generate material-specific volume fraction maps, depending on the scanner setup. Traditionally, two approaches were the most common: dual-source CT, which uses two distinct X-ray sources set to different kV; and fast kV switching [21,22,23]. In this study, we used a technique more recently introduced to the market, the dual-layer spectral CT, in which two different detector layers with different sensitivity peaks on the X-ray energy spectrum are stacked upon each other. By exploiting material- and energy-dependent X-ray absorption, the dual-layer spectral CT technique enables material decomposition and material-specific volume fraction measurements [22]. Compared to dual-energy CT, the advantage of spectral CT is that it allows for the collection and analysis of spectral information without the use of a predefined specific protocol (“always on”).

Several previous studies have shown the utility of DECT for detecting BME, all of which used MR imaging as standard of reference [7,24,25,26,27]. One study by Guggenberger et al. showed that DECT allows for detection of bone marrow lesions in acute ankle joint trauma with a sensitivity of 90% and specificity of 80.5% [25]. Another study found a sensitivity of 86.4% and specificity of 94.4–95.5% for the detection of BME in patients with acute knee trauma [24]. Further studies showed that DECT allows for the detection of BME in patients with vertebral fractures and therefore might help differentiate between acute and old vertebral fractures [7,8,26]. All of those studies showed that DECT might provide an alternative to MR imaging when it comes to the detection of BME. However, those studies were based on BME as a result of trauma.

In this study we demonstrated that three-material decomposition derived from dual-layer spectral CT may also be used for the detection of OO-associated BME, with a very high sensitivity of 92% and specificity of 94%. Those results are even more promising than those in the studies mentioned above. Additionally, the correlation between BME size measurements in MRI and DLCT-derived volume fraction maps was high. Interreader agreement for both Likert scale ratings and the measurements of the extent of BME in patients with OO were substantial in DECT images, indicating that our results are independent from the reader.

This study has limitations. The sample size assessed in this study was fairly small. Therefore, future studies with larger study cohorts are needed to confirm our findings. Furthermore, we only looked at BME in patients with OO and did not include patients with other pathologies also associated with BME. Therefore, the role of BME detection on DLCT for differentiating OO from other entities still needs to be evaluated. Additionally, the reading of material-specific volume fraction maps derived from dual-layer spectral CT images might be biased by the fact that the maps might also have shown the nidus, and therefore, the readers might more likely have rated the categories “3” and “4” in images with presence of a nidus. Another potential source of bias could be due to the fact that readers were allowed to adjust the window settings. As mentioned above, often the first imaging modality used was MR imaging, especially in young patients; therefore, the presented technique, which aims to replace the MR scan in addition to a CT scan, might not be useful in all patients. Yet, in patients with atypical clinical presentations with pain that does not respond to NSAIDs and where no obvious abnormalities on plain X-ray images are reported, MR imaging is often performed in order to investigate the underlying cause. However, in incidental findings on CT scans, the presence or absence of BME might support the clinical decision-making process and further clinical workup of certain patients. The parametrical maps can only be used qualitatively to distinguish between relative differences of water rich and fatty bone tissue. This can be explained by the presence of different materials within the region of interest, which are not taken into account by the three-material decomposition algorithm. This results in a bias in the volume fraction maps, especially in the water and fat maps. However, the differentiation of water rich tissue from fatty tissue by relative deviations within the investigated bone structure is still feasible analogous to the investigation of MR STIR maps. So far, DLCT for the detection of BME has not yet been evaluated in patients after therapy of OO and therefore should be subject of future studies.

## 5. Conclusions

In conclusion, this study showed that material-specific volume fraction maps derived from dual-layer spectral CT images allowed for the detection of BME in patients with OO. This may be of high clinical importance as it might spare patients with suspected OO an additional examination and facilitate the diagnosis of OO.

## Figures and Tables

**Table 1:** Localization of osteoid osteoma.

Localization	*n* = 18
Tubular bones: Epiphyseal	2
Metaphyseal	4
Diaphyseal	10
Spine: Vertabral body	1
Posterior elements	1
Subperiostal	14
Cortical	4
Intramedullar	0
Extra-articular	6
Intra-articular	14

**Table 2:** Diagnostic performance of dual-layer spectral CT over all patients for the detection of BME with MR imaging as standard of reference. Sensitivity, specificity, and accuracy are given with 95% confidence interval.

	Radiologist 1	Radiologist 2	Overall
Sensitivity	0.89 [0.64–1.00]	0.94 [0.69–1.00]	0.92 [0.72–1.00]
Specificity	1.00 [0.80–1.00]	0.89 [0.64–1.00]	0.94 [0.74–1.00]
Accuracy	0.94 [0.75–1.00]	0.92 [0.70–1.00]	0.93 [0.73–1.00]

## Data Availability

All required data is available within the manuscript.

## Abbreviations

Bone marrow edema	BME
Confidence interval	CI
Dual-layer computed tomography	DLCT
Intraclass correlation coefficient	ICC
Spectral base image	SBI
Osteoid osteoma	OO
